# Supplementary material for: A fault-intrusion-tolerant system and deadline-aware algorithm for scheduling scientific workflow in the cloud
Source: PeerJ Comput Sci. 2021 Nov 2;7:e747. doi: 10.7717/peerj-cs.747 (PMC8576554; doi:10.7717/peerj-cs.747)
Supplement: Supplemental Information 6 [file peerj-cs-07-747-s006.zip › Myproject1.1/docs/org/cloudbus/cloudsim/package-frame.html]

org.cloudbus.cloudsim


# org.cloudbus.cloudsim

## Interfaces

- *Packet*
- *Storage*
- *UtilizationModel*

## Classes

- Cloudlet
- CloudletScheduler
- CloudletSchedulerDynamicWorkload
- CloudletSchedulerSpaceShared
- CloudletSchedulerTimeShared
- Consts
- Datacenter
- DatacenterBroker
- DatacenterCharacteristics
- DataCloudTags
- File
- FileAttribute
- HarddriveStorage
- Host
- HostDynamicWorkload
- HostStateHistoryEntry
- InfoPacket
- Log
- NetworkTopology
- Pe
- ResCloudlet
- SanStorage
- UtilizationModelFull
- UtilizationModelNull
- UtilizationModelPlanetLabInMemory
- UtilizationModelStochastic
- Vm
- VmAllocationPolicy
- VmAllocationPolicySimple
- VmScheduler
- VmSchedulerSpaceShared
- VmSchedulerTimeShared
- VmSchedulerTimeSharedOverSubscription
- VmStateHistoryEntry

## Exceptions

- ParameterException
